# Who votes when? Midterm and presidential voters differ primarily by age

**DOI:** 10.1371/journal.pone.0360051

**Published:** 2026-10-14

**Authors:** Brian T. Hamel, Jan E. Leighley, Jonathan Nagler

**Affiliations:** 1 Department of Political Science, University of North Texas, Denton, Texas, United States of America; 2 Department of Government, American University, Washington, District of Columbia, United States of America; 3 Wilf Family Department of Politics, New York University, New York, New York, United States of America; University of Stavanger, NORWAY

## Abstract

Voters in American congressional midterm elections are widely viewed as demographically less representative than those in presidential elections – older, whiter, wealthier, and more educated. Using Current Population Survey data from 1994 through 2024 as well as voter-file data, we test this conventional wisdom and show that it is partly right but too broad. The key difference between midterm and presidential electorates is age: midterms sharply underrepresent young citizens and overrepresent older citizens compared to presidential elections, while differences between the elections by race, income, and education are much smaller. Midterm voters are, above all, older voters.

## Introduction

Voters are not a representative cross-section of American citizens. Compared with nonvoters, voters tend to be older, wealthier, more educated, and whiter; they are also more conservative on questions of redistribution and the role of government [1,2]. These disparities matter because unequal participation can translate into unequal political voice: groups that are overrepresented among voters may exercise greater influence over electoral outcomes and public policy [3–6].

A longstanding expectation is that lower-turnout election produce greater inequalities in who votes [7–10]. The logic is straightforward: when overall participation declines, citizens with greater material and informational resources are less likely to drop out of the electorate. Because their participation is comparatively resilient, they comprise a larger share of voters in lower-turnout elections than they do when turnout is higher. Lower-turnout elections may therefore give even more disproportionate political voice to more advantaged groups.

U.S. congressional midterms offer a particularly useful setting in which to evaluate this argument. Midterm and presidential elections draw from the same underlying population of eligible voters, but midterms are less salient, generating substantially less campaign activity, media coverage, and participation. Indeed, in recent years, turnout among the voting-eligible population has been roughly two-thirds in presidential elections but less than half in midterm elections [11]. If lower turnout broadly magnifies demographic inequalities, midterm electorates should be less representative of the citizen population than presidential electorates across multiple dimensions. Popular press commentary reflects this expectation, routinely portraying midterm voters as older, whiter, wealthier, and more educated than presidential voters [12–17].

Yet this broad expectation has not been evaluated systematically across demographic characteristics and over multiple election cycles. Much of the available evidence is limited to individual elections [18], making it difficult to know whether any observed pattern is durable or specific to the electoral context in which it arises. It is also unclear whether lower-turnout elections broadly intensify demographic inequalities in representation or primarily amplify inequalities along certain dimensions while leaving others largely unchanged. There are theoretical reasons to expect variation across different demographic groups. In particular, younger citizens have had fewer opportunities to develop the habit of voting [19,20], which may make their participation especially sensitive to the lower salience of midterm elections. Age-based inequality may therefore widen more sharply than other forms of demographic inequality.

Existing analyses also tend to rely on group-specific turnout rates [21], which do not directly indicate a group’s electoral weight relative to its presence in the population. For example, suppose 60% of one demographic group votes. Whether that group is over- or underrepresented in the electorate depends not on that turnout rate alone, but on how it compares with turnout in the population. If overall turnout is 70%, the group will be underrepresented among voters; if overall turnout is 50%, the same group will be overrepresented. Group-specific turnout rates therefore tell us how likely members of a group are to vote – but on their own cannot directly answer questions of representational equality.

We examine the magnitude, sources, and stability of midterm representativeness across three decades of U.S. federal elections using the Current Population Survey (CPS) Voting and Registration Supplement from 1994–2024 and voter-file data from Catalist for 2008–2024. We compare the demographic composition of presidential and midterm voters with that of the adult citizen population. Our central measure is a *representation ratio*: each group’s share of voters divided by its share of the adult citizen population. Unlike group turnout rates, the ratio captures whether a group is over- or underrepresented among those who participate, providing a common metric for assessing representational inequality across demographic characteristics and election types. Our analysis examines whether midterms disproportionately under- or overrepresent citizens by age, education, gender, income, and race; the size of those disparities; and their stability over time.

We find that age is the demographic dimension that most consistently differentiates midterm and presidential voters. Theoretically, our findings suggest that the effects of lower turnout on representational inequality depend not simply on resources or social advantage, but on the particular mechanisms through which group characteristics shape participation across electoral contexts. In closing, we briefly examine partisan and policy preferences across age groups to assess whether these differences may have politically meaningful consequences in midterms, and highlight directions for future research on the mechanisms underlying the disproportionate decline in youth representation from presidential to midterm elections.

## Materials and methods

Our primary analysis uses the Current Population Survey Voting and Registration Supplement for federal elections from 1994 through 2024. The pooled sample includes nearly 1.5 million voting-age citizens across sixteen elections, with survey weights for the U.S. civilian noninstitutionalized population. We restrict the sample to U.S. citizens ages 18 and older.

The CPS measures turnout through self-reports, which are susceptible to over-reporting bias [22–25]. The Census Bureau also conventionally codes respondents who do not answer the turnout question as nonvoters. Following Hur and Achen [26], we treat these cases as missing and implement their recommended state-level weight adjustment: within each state, we rescale the weights of self-reported voters and nonvoters so that weighted turnout matches the corresponding external state-level turnout benchmark.

Our primary quantity is the representation ratio for demographic group *g*:


$$\begin{array}{c} {RR}_{g}=\;\frac{\text{Pr}(G=g\;|Voted)}{\text{Pr}(G=g)} \end{array}$$

The numerator is the weighted share of voters belonging to group *g*, and the denominator is the weighted share of adult citizens belonging to that group. Values greater than 1 indicate overrepresentation among voters relative to the adult citizen population, whereas values below 1 indicate underrepresentation.

We calculate representation ratios separately for each demographic group, election type, and election year. We examine differences across five key demographic characteristics. Four – age, education, income, and race – are commonly identified in journalistic accounts as dimensions along which midterm elections are especially unrepresentative relative to presidential elections. We also look at gender. Age is grouped as 18-29, 30-44, 45-64, and 65 or older. Education is grouped as less than a high school education, high school graduate, some college, and a bachelor’s degree or higher. Gender is measured as male or female. Income groups are weighted within-year terciles constructed from ordered CPS family-income categories. Race is measured as non-Hispanic White versus non-White. Unlike age, education, and income, racial categories do not have a natural ordering, and a more disaggregated coding would require choosing among numerous pairwise comparisons without a clear theoretical basis for privileging one over another. We therefore use this broad distinction to summarize racial representational inequality.

To examine temporal stability in midterm bias, we calculate the *gap* in representation ratios between groups at opposite ends of the relevant comparison. For age, education, and income, which have a natural ordering, we define the gaps as ages 65 or older minus ages 18-29, college degree or higher minus less than a high school education, and high income minus low income. The endpoint approach focuses on differences between the most advantaged and disadvantaged categories of each ordered demographic characteristic rather than incorporating all intermediate categories. These comparisons correspond to common substantive distinctions in political research and discourse, while the discrete categorical structure of the data makes a continuous inequality measure, such as a Gini coefficient, less appropriate. Gender and race have only two categories, so we define the corresponding gaps as female minus male and non-Hispanic White minus non-White, respectively. We then subtract the representation gap in the preceding federal election from the gap in the current election for each demographic category, indicating whether representational inequality widened or narrowed from one election to the next. Positive values indicate that the gap widened, and negative values indicate that it narrowed.

We replicate our temporal CPS analysis using publicly available Catalist voter composition data from 2008 through 2024. Catalist is a private-sector voter database and data analytics firm that works with progressive organizations and campaigns. Its public reports provide demographic shares of voters based on voter file data for characteristics contained in the voter file (such as age), and proprietary modeling for characteristics not included in the voter file (such as education). We calculate representation ratios using these shares as the numerator and American Community Survey (ACS) estimates of each group’s share of the citizen voting-age population as the denominator. The replication includes age, education, gender, and race; Catalist does not report voter composition by income. Catalist classifies education as college versus non-college. Thus, the education analysis is not an exact replication of the CPS analysis, which uses four education categories and calculates the representation gap between respondents with less than a high school education and those with at least a college degree.

Finally, to illustrate the potential political consequences of age differences in voter composition, we use the 2022 and 2024 Cooperative Election Studies, large national online surveys administered by YouGov using a nonprobability sample matched and weighted to approximate the demographic composition of the U.S. adult population. We calculate weighted support within each age group on six salient issues plus partisan identification and assess the potential electoral consequences of age-based midterm bias using a House vote counterfactual that reweights the 2022 electorate to match the age distribution of voters in the 2020 presidential election.

## Results

Fig 1 pools data across all federal election years from 1994 through 2024 and reports representation ratios separately for presidential and midterm elections. Across both election types, voters are older, wealthier, more educated, and whiter than the adult citizen population; the voter pool is, however, roughly balanced by gender. The central question is whether midterms substantially intensify the familiar patterns of age, education, income, and racial bias relative to presidential elections.

**Fig 1:**
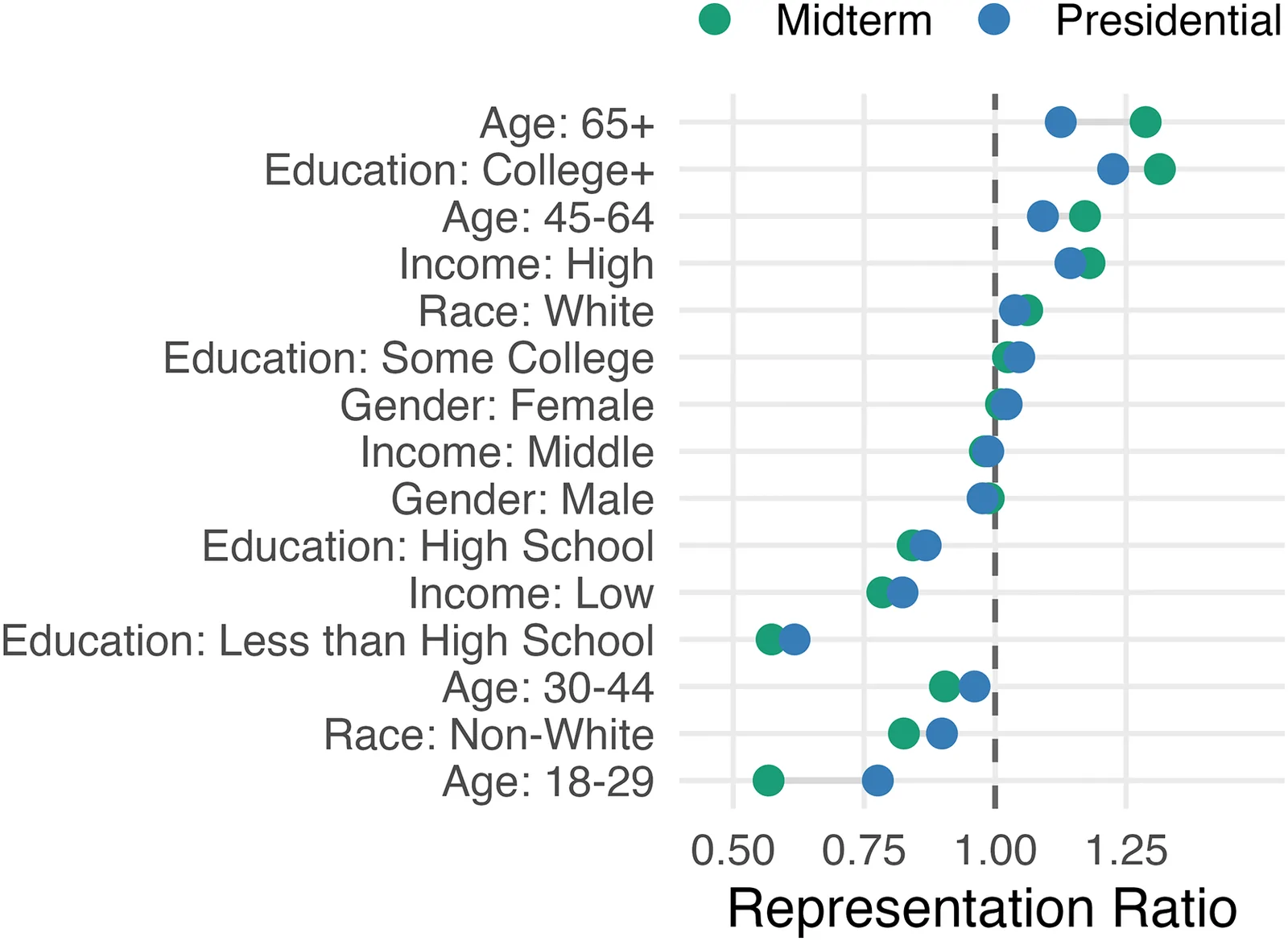
Representation ratios by demographic group and election type, Current Population Survey Voting and Registration Supplement, 1994-2024. Values above 1 indicate that a group is overrepresented among self-reported voters relative to its share of the adult citizen population; values below 1 indicate underrepresentation.

The largest and clearest difference in representation ratios between midterm and presidential elections is for age. Adults ages 18-29 have a representation ratio of 0.57 in midterms – meaning that their share of voters is only 57% of their share of the adult citizen population – compared with 0.78 in presidential elections. In even more concrete terms, if 18-29-year-olds made up 20% of the adult citizen population, a representation ratio of 0.57 would mean that they accounted for only about 11% of voters in midterm elections. By comparison, a representation ratio of 0.78 in presidential elections would mean that they accounted for about 16% of voters. Adults ages 65 and older exhibit the reverse pattern: their representation ratio rises from 1.13 in presidential elections to 1.29 in midterms. Put differently, presidential electorates already underrepresent younger adults and overrepresent older adults, but these disparities become substantially larger in midterms.

The patterns differ across the other demographic categories. Gender, race, and income show relatively little change in representational inequality between presidential and midterm elections. Education-based inequality is substantial in both election types, but unlike age, it does not widen much in midterms. Indeed, education is the largest source of representational inequality in presidential elections, whereas age becomes the largest in midterms. The distinction is therefore not that age is the only source of demographic inequality in American electorates. Rather, age is the dimension on which midterm elections most sharply amplify existing inequalities in representation.

Fig 2 examines whether this pattern persists over time by plotting changes in demographic representation gaps between the most and least advantaged group within each demographic category from one federal election to the next. Recall that positive values indicate that a gap widened relative to the preceding election, whereas negative values indicate that it narrowed. The age gap follows a pronounced electoral cycle: it widens in each midterm election and narrows again in the following presidential election. Changes in representation gaps are much smaller for education, gender, income, and race. Indeed, election-to-election changes in the age gap are generally greater than 0.25–roughly twice as large as the changes observed for other demographic characteristics.

**Fig 2:**
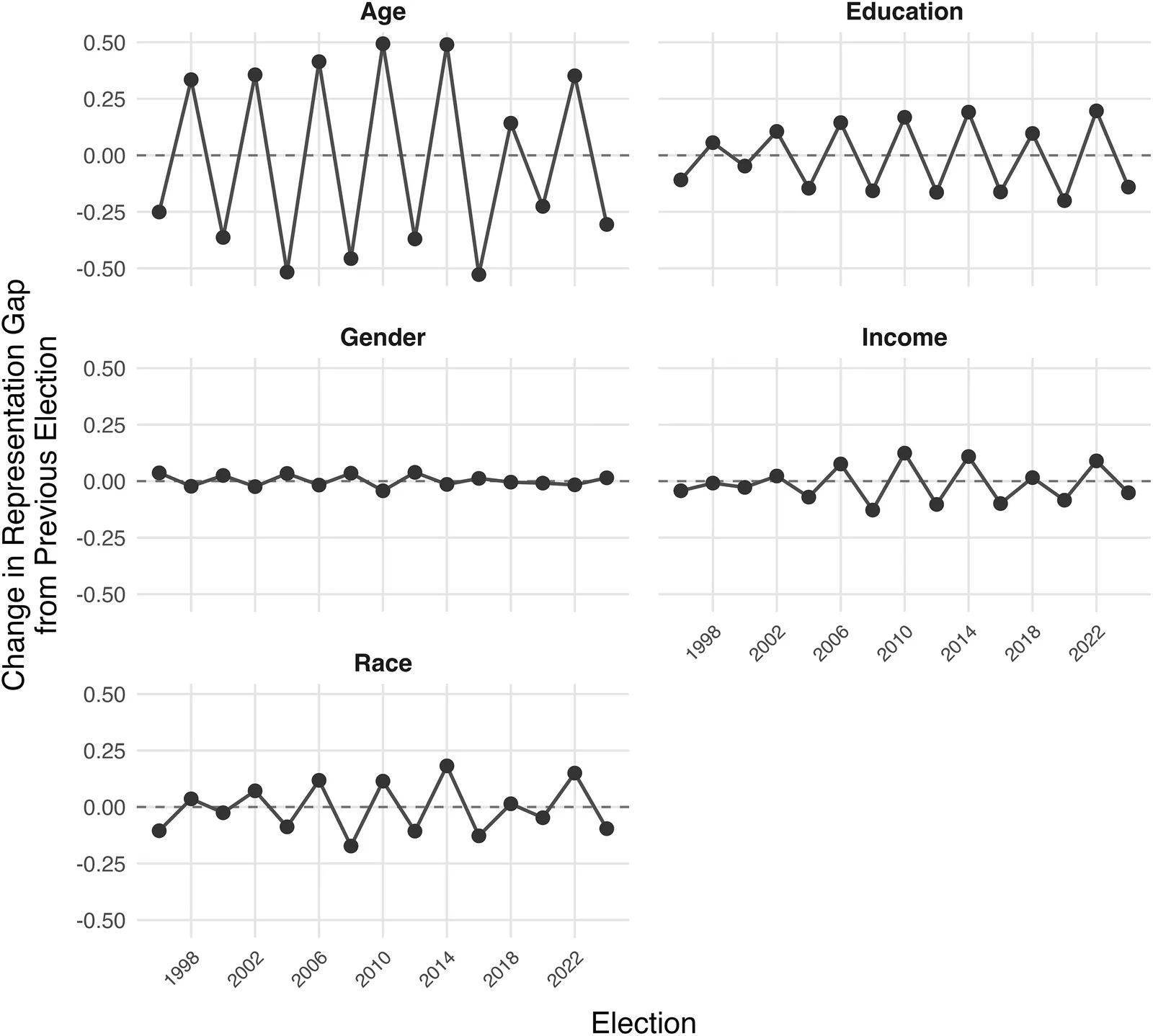
Change in demographic representation gaps from one federal election to the next, Current Population Survey Voting and Registration Supplement, 1994-2024. Positive values indicate that the gap between the most represented and least represented group widened relative to the previous election; negative values indicate that it narrowed.

Fig 3 replicates this temporal analysis using Catalist voter-file estimates of voter composition from 2008 through 2024. The same pattern emerges: age exhibits the largest and most consistent differences across election types, with representation gaps increasing sharply in midterms and declining in presidential elections. The convergence of the CPS and Catalist results indicates that the central finding is not an artifact of survey-based measurement.

**Fig 3:**
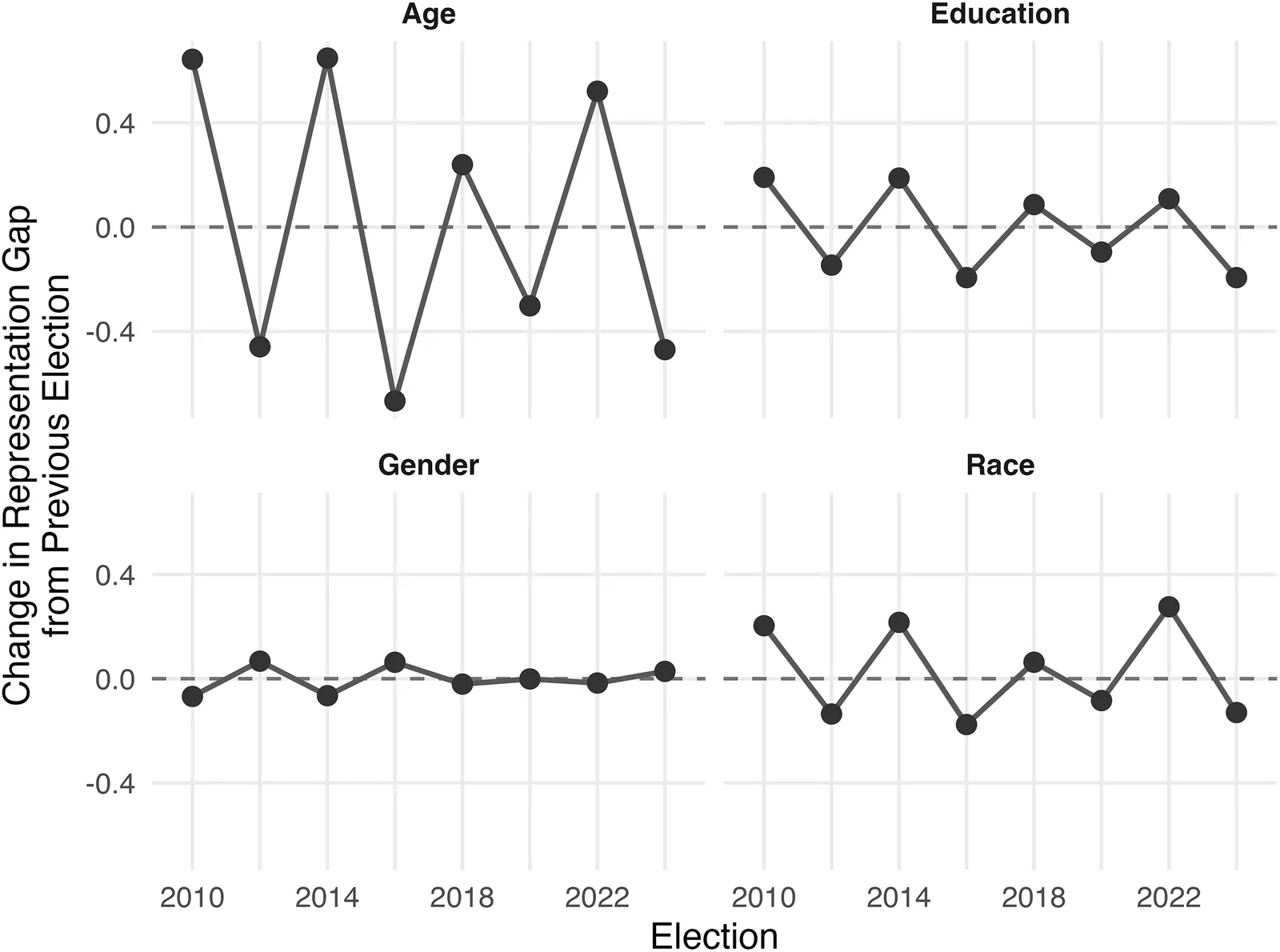
Change in demographic representation gaps from one federal election to the next, Catalist, 2008-2024. Positive values indicate that the gap between the most represented and least represented group widened relative to the previous election; negative values indicate that it narrowed.

## Discussion

Our findings clarify the nature of midterm bias: midterm elections are less representative than presidential elections, but not equally so across demographic dimensions. Across three decades, the over-representation of White, higher-income, and more educated voters is substantial in both election types and changes relatively little between them. By contrast, age-based inequality is consistently much greater in midterms than in presidential elections: older voters are more over-represented in midterms while younger voters are much more under-represented in midterms. The most striking representational feature of midterm elections is therefore the relative disappearance of young voters: they simply stay home at much higher rates in midterms than they do in presidential elections. Midterm voters are, above all, older voters. More broadly, our results qualify the common assumption that lower turnout necessarily magnifies demographic bias across the board. Moving forward, scholars and journalists should be sure to distinguish persistent participation biases from the specific forms of bias that midterms intensify.

By participating at lower rates in midterm elections, younger voters have less ability to influence who is in office and, potentially, what policies are adopted by the government. Such disparities in political influence may have especially significant consequences because younger and older Americans differ in both partisan identification and preferences on key issues [27–29]. Our own analysis of the 2024 Cooperative Election Study (CES) confirms these differences. Fig 4 shows that younger Americans are somewhat more Democratic than older Americans: 47% of those ages 18-29 identify with or lean toward the Democratic Party, compared with 42% of those ages 65 and older. It also shows that support for a U.S.-Mexico border wall, increased police funding, and repeal of the Affordable Care Act rises with age, while support for abortion rights, expanding Medicaid eligibility, and investment in renewable energy declines. When the midterm electorate is especially skewed toward older Americans, it therefore gives greater weight to the positions they are more likely to hold – positions that, as we have shown, are generally more conservative.

**Fig 4:**
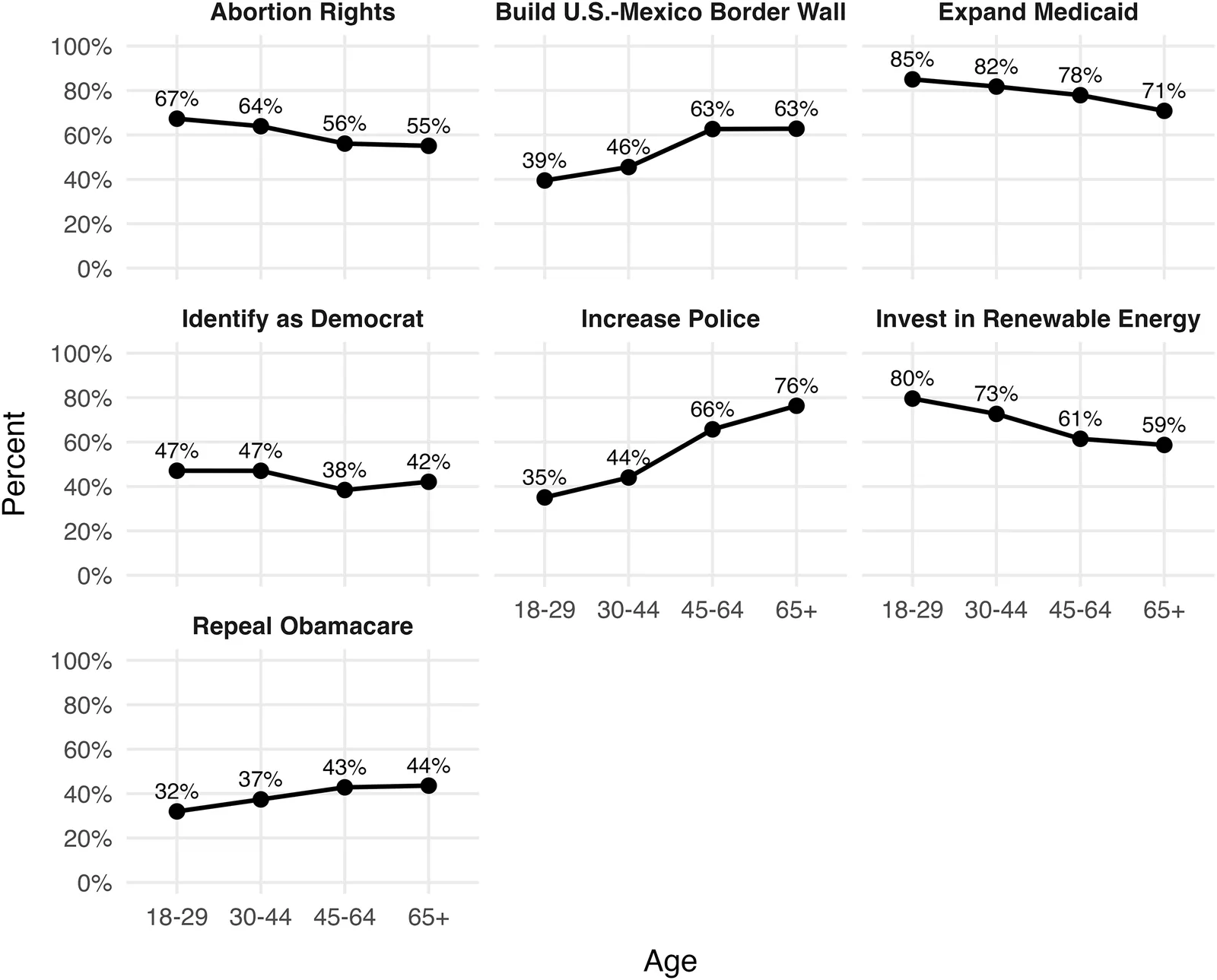
Partisan and policy preferences by age group, Cooperative Election Study, 2024. Points show the weighted percentage of respondents in each age group identifying as Democratic or supporting each policy position.

We can assess the electoral consequences of this age imbalance more directly by asking how the 2022 national U.S. House vote would have changed if the midterm voters had matched the age distribution of the voters in the preceding presidential election. Using the 2022 CES, we estimate the two-party Democratic House vote share among validated voters in each age group. We then combine those age-specific vote shares using Catalist’s age distribution of voters in the 2020 presidential election. Holding age-specific vote choice fixed, this produces a counterfactual two-party Democratic share of the national House vote of 50.1%–an increase of 1.5 percentage points relative to the actual 2022 two-party Democratic House vote share. The distinctive age composition of the midterm electorate can therefore have meaningful consequences for the national electoral balance, particularly in closely divided elections where relatively small shifts in vote share may affect which party controls the House.

An important question for future research is why age, more than the other demographic characteristics we examine, is especially susceptible to midterm bias. Several mechanisms may contribute. As noted, younger citizens may be less politically socialized into regular voting, and these life-cycle differences may make their participation more sensitive to election salience and mobilization. Younger citizens may also perceive midterm elections as less consequential than presidential elections; lower levels of news coverage and campaign activity may reinforce this perception and further discourage participation. Distinguishing among these explanations is beyond the scope of our analysis but is an important direction for future work. Comparative research could also examine whether the pronounced age bias we observe is distinctive to the institutional structure of U.S. elections or emerges in other political systems when comparing elections of different levels of salience.

One open question is whether concerted efforts by campaigns or civic organizations to mobilize young voters can narrow turnout gaps and related inequalities in representation. Even when such efforts succeed in a given cycle, our analysis suggests that sustaining those gains may be difficult. As Figs 2 and 3 show, age-based underrepresentation declined substantially in the 2018 midterm relative to earlier midterms. The change did not persist, however: by 2022, the age gap had returned close to levels observed in previous midterms. The 2018 case therefore suggests that even meaningful progress in a single cycle may not produce a lasting shift in the composition of the midterm electorate.
